# RETRACTION: Circ_0087199 Depletion Attenuates Lipopolysaccharides‐Induced Human Periodontal Ligament Cell Injury Through the miR‐527/TLR4 Axis

**DOI:** 10.1002/iid3.70219

**Published:** 2025-06-27

## Abstract

**RETRACTION:** J. Wang, N. Huo, C. Cai, Y. Zhang and R. Xiao, “Circ_0087199 Depletion Attenuates Lipopolysaccharides‐Induced Human Periodontal Ligament Cell Injury Through the miR‐527/TLR4 Axis,” *Immunity, Inflammation and Disease* 12, no. 1 (2024): e1153, https://doi.org/10.1002/iid3.1153.

The above article, published online on 24 January 2024 in Wiley Online Library (wileyonlinelibrary.com), has been retracted by agreement between the journal Editor‐in‐Chief, Marc Veldhoen; and John Wiley & Sons Ltd. The retraction has been agreed upon due to the unclear rationale for the study and the inability of readers to reproduce the results based on the information provided in the article. Despite the authors’ claim that the data would be available upon request, they did not respond to our request for the data. The editors consider the results and conclusions unreliable. The authors were informed of the decision to retract but did not respond to requests for comment.

**RETRACTION:** J. Wang, N. Huo, C. Cai, Y. Zhang and R. Xiao, “Circ_0087199 Depletion Attenuates Lipopolysaccharides‐Induced Human Periodontal Ligament Cell Injury Through the miR‐527/TLR4 Axis,” *Immunity, Inflammation and Disease* 12, no. 1 (2024): e1153, https://doi.org/10.1002/iid3.1153.

The above article, published online on 24 January 2024 in Wiley Online Library (wileyonlinelibrary.com), has been retracted by agreement between the journal Editor‐in‐Chief, Marc Veldhoen; and John Wiley & Sons Ltd. The retraction has been agreed upon due to the unclear rationale for the study and the inability of readers to reproduce the results based on the information provided in the article. Despite the authors’ claim that the data would be available upon request, they did not respond to our request for the data. The editors consider the results and conclusions unreliable. The authors were informed of the decision to retract but did not respond to requests for comment.

